# Human babesiosis: The past, present and future

**DOI:** 10.1017/erm.2025.10016

**Published:** 2025-09-05

**Authors:** Madison Asquith, Sally Prior, Anke Brüning-Richardson

**Affiliations:** School of Applied Sciences, University of Huddersfield, Huddersfield, UK

**Keywords:** babesia, babesiosis, diagnosis, epidemiology, treatment, vector, zoonosis

## Abstract

Human babesiosis is a disease transmitted by the bite of an infected tick or via blood transfusions involving contaminated blood products; in humans, it can lead to severe complications and even death, depending on the clinical history, age and health status of the affected patient. Babesiosis is caused by members of the *Babesia* spp., protozoan parasites whose life cycle includes sexual reproduction in the arthropod vector and asexual reproduction in the mainly mammalian host. Cases of human babesiosis have been rare, but there are increasing reports of human babesiosis associated with climatic changes affecting the geographical distribution of the parasite and tick vector, enhanced vector–human interactions and improved awareness of the disease in humans. Diagnostics and treatment options for humans are based around discoveries in veterinary research, such as point-of-care testing in cases of bovine babesiosis, and include direct diagnosis by blood smears, polymerase chain reaction (PCR) and enzyme-linked immunosorbent assay (ELISA) technologies, and indirect diagnosis by ELISA, immunofluorescence tests (IFAT) and fluorescent in situ hybridisation. Treatment involves a combination of drugs such as azithromycin and atovaquone, or clindamycin and quinine, but more effective options are being investigated, including, but not limited to, trans-chalcones and tafenoquine. Improved surveillance, awareness and diagnosis, as well as advanced technologies to interrupt vector–host interactions, are crucial in managing the increased threat posed by this once-neglected disease in humans.

## Introduction

Changes in climate, habitats and human lifestyles have had a major impact on the epidemiology and expansion of parasites of both medical and veterinary importance beyond their tropical confinements (Refs. 1–3). These phenomena are also observed with parasites such as malaria, where rises in temperature have recently been directly correlated with an increase in recorded cases in the United Kingdom (Ref. 4). Similarly, akin parasites of medical importance previously associated with or confined to tropical regions, that also rely on blood-sucking vectors, include *Babesia* (Ref. 5). These blood parasites affect animals and birds and can also be transmitted to humans via the bite of a tick vector or contaminated blood products to cause a disease known as babesiosis. Although once a neglected disease in humans due to low transmission rates, cases in humans have been steadily increasing over the last 20 years (e.g., from 2011 onwards) (Ref. 6). This review provides an update on the impact of babesiosis on human health in the past, present and future, with a particular focus on novel ways to diagnose and treat these zoonotic diseases, while also uncovering the research areas that require further investigation.

### *Babesia* spp

Babesiosis is a mostly zoonotic disease caused by infection with parasite species of the genus *Babesia*, which are haemoparasites that are found globally, particularly in tropical and subtropical regions (Ref. 7). The parasites are transmitted by ixodid ticks as part of their life cycle, with the parasite undergoing sexual replication in the vector and asexual replication in the (mainly) mammalian host. Although the parasites preferentially infect an animal host, some can also be transmitted to humans, making it a zoonotic disease. The resulting disease causes serious medical, veterinary, and associated economic burdens worldwide and can be fatal to humans, particularly in the elderly, the young, and immunocompromised, with a mortality rate of around 20% (Ref. 8). Babesiosis and its causative agent, *Babesia*, were named after the microbiologist Viktor Babes in his honour, who first identified bacteria-like organisms in the red blood cells (RBCs) of cattle that exhibited bouts of haemoglobinuria, the presence of haemoglobin in urine (Ref. 9). Haemoglobinuria is a characteristic of the disease, causing a distinctive reddening of the urine (which can vary from a shade of rose to black) in an infected animal. The physician Theobald Smith and veterinarian Fred Kilbourne discovered the role of ticks as disease vectors of babesiosis in 1893, marking the first recorded case of protozoan parasite transmission by an arthropod (Refs. 10–11). In the same year, *Babesia* was further divided into distinct species: *Babesia bovis*, *Babesia ovis* and *Babesia bigemina (*all species affecting cattle or sheep), by protistologist Constantin Starcovici (Refs. 12–13). The first human case of babesiosis was recorded in 1956, when *Babesia divergens* (retrospectively presumed) was discovered in the blood and bone marrow of a splenectomised patient who presented with haemoglobinuria (Ref. 14). *Babesia* is a parasite commonly found in free-living/domestic animals, attributed to the ubiquitous nature of tick vectors and the parasites’ distinct mode of transmission (Ref. 15). Previously underreported, *Babesia* has been described as an emerging pathogen in humans in recent years, with increasing numbers of cases reported globally, from both traditional transmissions, along with blood-transfusion transmitted cases, also spread via organ-transplantation or maternal-foetal transmission, therefore requiring efficient diagnosis and effective treatment options to prevent its continued spread (Ref. 8).

*Babesia* spp. present as unicellular, pear-shaped forms (1–5 μm in length) inside erythrocytes of infected hosts, including humans, capable of infecting a variety of vertebrate hosts to maintain its cycle of transmission. There are over 100 species of *Babesia*, but only several of these are known to infect humans, including *B. crassa-*like*, B. divergens, B. duncani, B. microti, B. venatorum*, *B. odocoilei, B. bovis* and *B. bigemina*, although new species periodically emerge as transmissible to humans. Infection of *Babesia* in livestock presents a serious economic problem worldwide, caused by species such as *B. bigemina, B. bovis* and *B. divergens* and so forth. *Babesia* may also cause infection in other animal species, such as horses (*B. caballi and B. equi* (which is now classified as *Theileria equi*)) and dogs (*B. canis*) (Ref. 16).

**Figure 1.**
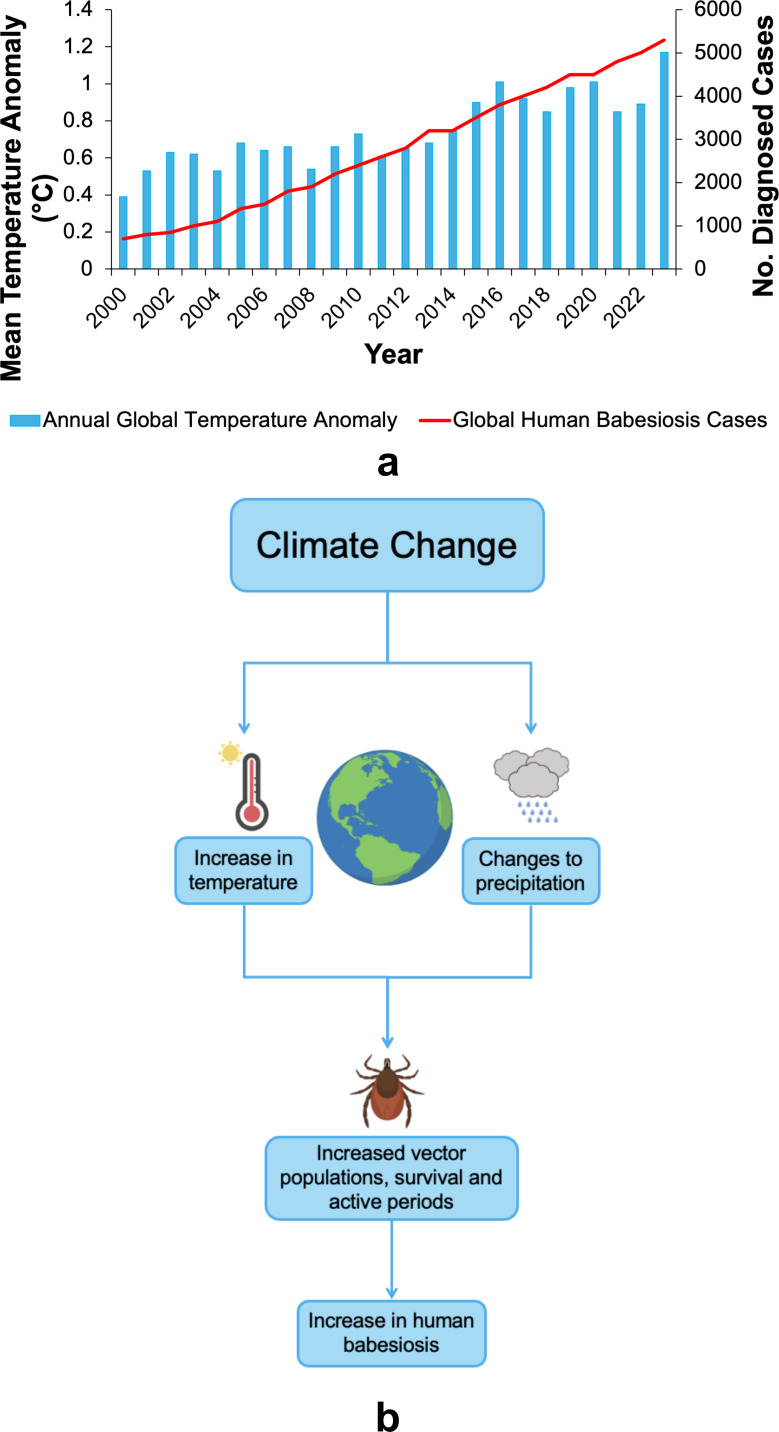
Climate change is correlated with the increasing emergence of human babesiosis. (a) Global human babesiosis cases increase in line with the mean annual temperature anomaly, calculated by comparing the average temperature from 1951 until 1980, shown in blue (Refs. 23–27). Data on human babesiosis cases, plotted in red, were combined from a range of databases containing estimates on the number of diagnosed cases per year. (b) A diagram showing the effects of climate change on human babesiosis, where increased temperature and differences in rainfall patterns result in larger vector populations, enhanced vector survival and longer periods of vector activity.

It is thought that climate change could contribute to the emergence and spread of *Babesia* vectors through the gradual increase in global temperature (e.g., from 2000 onwards), thereby directly impacting how human babesiosis is distributed (e.g., in the United States) (Figure 1a) (Refs. 17–19). The warmer global temperature is believed to impact vectors involved in *Babesia* transmission, such as increasing vector survival and their populations, along with increasing the length of time periods where they are most active (Figure 1b) (Refs. 20–21). Examples of this spread have already been observed, such as in North America (Ref. 22). This increase can also be attributed to an increased awareness of human cases of babesiosis and associated enhanced detection in a human host.

## Epidemiology

### Global distribution and trends

Alongside climate change, the occurrence of cases of babesiosis is also influenced by the geographical location of the parasites. Geographical distribution of *Babesia* varies greatly between species, but in general the highest prevalence of *Babesia* is in tropical and subtropical regions, such as the Middle East, Europe (42 cases in 2020), the Caribbean and especially Central/South America (24,386 cases in 2020), where babesiosis (typically bovine/equine) is considered endemic. This may be attributed to the warmer climate, where *Babesia* and, in particular, its vectors can thrive (Refs. 28–29).

The most common species seen in humans is *B. microti*, especially observed in the United States, but recent reports also state that *B. microti* has been detected in ticks and humans in Europeans countries, such as Germany (one case in 2020) and Poland (one case in 2020). *B. microti* in particular is known to be endemic in the northeastern and northern midwestern regions of the United States. Larger populations of *B. divergens* (26 out of 41 cases in 2020) and *B. venatorum* (4 out of 41 cases in 2020) are more common for Europe; however, a *B. divergens-*like genotype has also been reported in the United States, adding to its expanding association with human populations (Refs. 29–30).

Reporting of human cases started in earnest in 2011 (in the United States), where a drastic increase in cases per year has been observed since (a 25% increase from 2011 to 2019); however, cases are thought to be even higher due to a lack of reporting in asymptomatic patients (Ref. 31). Overall increase (not specifically of *B. microti*) appears to be multifactorial and involves (a) an increase in the population of white-tailed deer, which can act to amplify the population of *Ixodes* ticks, (b) an increase in human populations in woodland areas, (c) human encroachment of animal-vector environments, and/or (d) improved diagnosis and recognition/awareness of human babesiosis by medical professionals (Refs. 32–33). Interestingly, white-tailed deer are known to be reservoirs for various tick-borne, bacterial and viral pathogens, playing a key role in the transmission of zoonotic diseases to humans (Refs. 34–35). Alternatively, the natural reservoir for *B. microti*, in particular, is the white-footed mouse; however, each stage in the life cycle involves a different vertebrate host (from small mammals/birds for the larvae, to larger mammals for the adult ticks) (Ref. 36).

### US-specific *Babesia* cases

Large numbers of human cases are consistently reported in the United States (16,456 cases reported between 2011 and 2019) (Ref. 6). From 2014 (1,744 cases), the number of cases grew, with fluctuations, reaching a high of 2,418 in 2019 and then decreasing to 1,827 cases in 2020, potentially due to higher awareness (Ref. 24). According to geographic modelling, cases are expected to continue to rise (Ref. 8). Considering infections caused by blood transfusions, the Food and Drug Administration (FDA) in the United States now recommends that donors should be screened for *Babesia* spp. in United States where babesiosis is endemic, with the use of polymerase chain reaction (PCR) assays. This has served to prevent transfusion-transmitted babesiosis (TTB) cases, which were previously seen to be increasing in incidence in the United States (Ref. 37). In response to screening of blood samples, cases of TTB appear to be now on the decrease (as of 2023), with only 3 TTB cases reported after the recommendations were put in place (in the space of 2 years) in comparison to 44 cases in the 3 preceding years (Ref. 38).

### Recent global *Babesia* cases

**Figure 2.**
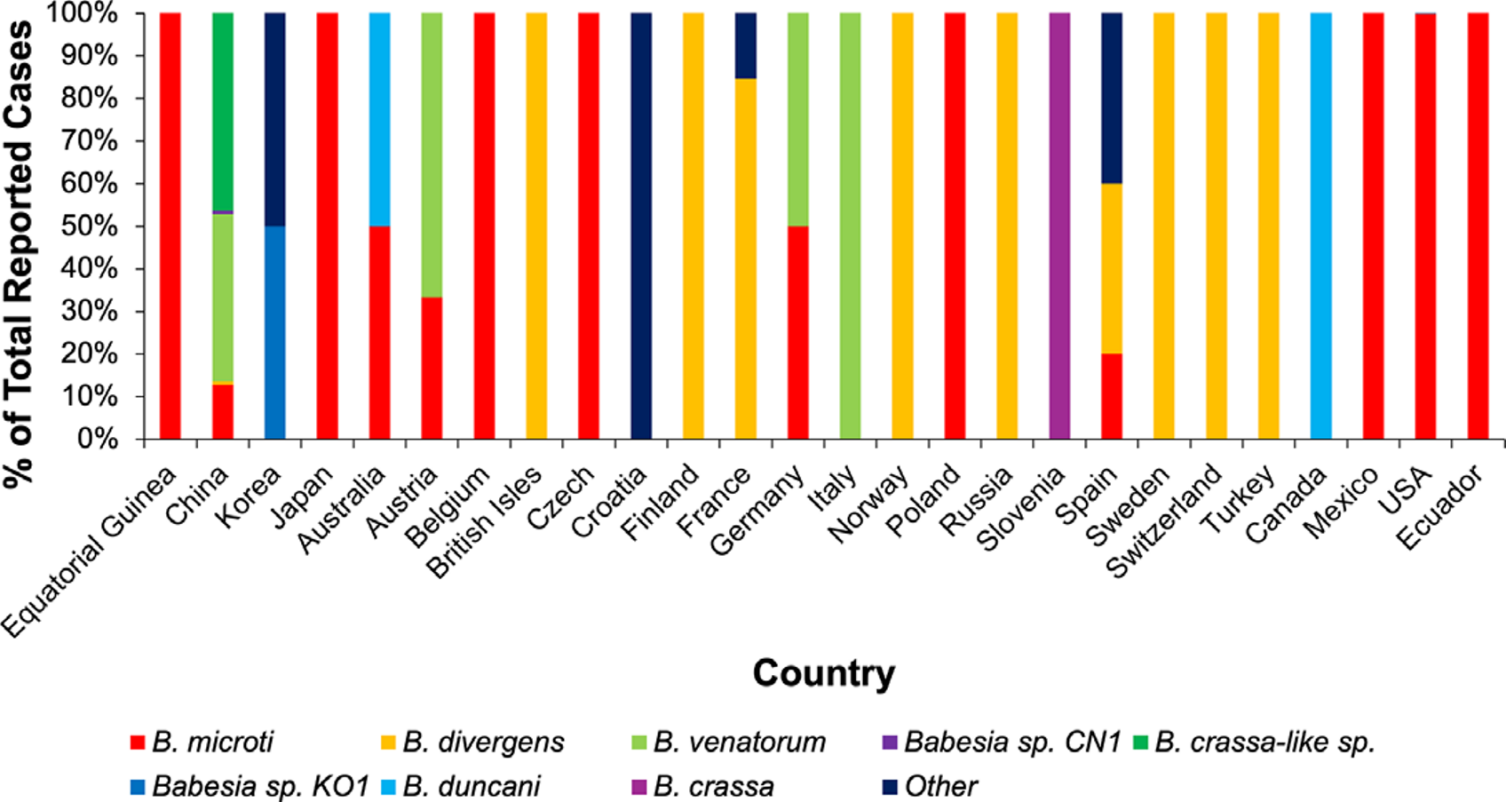
The species distribution of *Babesia* is associated with geographical location. Data from 2020 demonstrates the range of *Babesia* spp. detected, with countries such as Japan, Mexico and the United States reporting only *B. microti* infections, while *B. divergens* and *B. venatorum* are more prevalent across Europe. China detected the most *Babesia* spp. and the only cases of both *Babesia* spp*. CN1* and *B. crassa-like* spp. globally (Ref. 28).

Apart from the majority of cases recorded in the United States, globally, babesiosis cases appear to be on the rise (e.g., since 2020 onwards), with cases also detected in the United Kingdom. The United Kingdom saw its first case of babesiosis in 1979 in Scotland, with other cases seen in Ireland in the past, but the first case in England was recorded in 2020, according to Public Health England (Refs. 39–40). Residents have since been warned to stay vigilant and aware of ticks and the possible repercussions of being bitten (Ref. 40). In other parts of Europe, human babesiosis is mainly caused by *B. divergens*, *B. venatorum* and *B. microti*, whereas infections in Canada and the United States are predominantly caused by *B. duncani* and *B. microti*, respectively. In Asia, China appears to display the widest range of *Babesia* spp., with *B. microti*, *B. divergens*, *B. venatorum*, *Babesia sp. CN1* and *B. crassa-like* species detected in 2020. Species of *Babesia* such as *Brucella microti, B. divergens*, and *Bombylius duncani* have been noted as emerging zoonotic pathogens globally, along with other lesser-known species (Figure 2). There is evidence of human babesiosis caused by *B. odocoilei*, previously reported in animals (e.g., white-tailed deer, reindeer etc.), but not in humans (Refs. 41–43). Along with this, the discovery of new human-infectious *Babesia* spp. suggests the parasite is evolving, and research must follow this evolution, progressing diagnosis and treatment.

### Economic impact of babesiosis

Babesiosis has been described as an emerging disease, rising in cases over the years (e.g., 2011 onwards), in turn causing a huge economic burden globally, affecting not only the livestock industry but also gaining interest as a zoonotic disease in terms of vaccination, diagnosis and treatment of humans infected with *Babesia.* It follows that, as *Babesia* affects livestock, infections and resulting disease are especially hard on those who maintain these animals in socio-economically deprived countries, leading to financial strain in terms of vaccinations and treatment. The major economic impact of babesiosis is related to its burden on the dairy industry. It has been estimated that the disease causes losses of ~38.9 million dollars per year in Argentina, considering the loss of livestock, the cost of vaccines, treatments and associated administrative costs (Ref. 44). Globally, the economic burden is not entirely ascertainable, but a study in Tanzania determined this as approximately $50 million a year from 2006, attributed to lack of commercial dairy, pastoral and agro-pastoral production, treatment of infection, mortality of livestock and prevention (removal of vectors through acaricides) (Refs. 44–45).

There is a lack of research on the global economic burden of babesiosis in relation to humans, with only 0.5% of all articles describing economic impact (Ref. 17). Most cost analysis research originates from the United States and typically focuses on one area, such as the cost of laboratory diagnostic testing, rather than focussing on the global impact (Ref. 46). Research does suggest, however, that the main economic impact associated with human health is related to the cost of diagnosis and treatment for symptomatic human babesiosis cases in the United States, Japan, Italy and so forth (Ref. 17). Further studies should be completed to determine the full global impact of human babesiosis, including the cost of treatment and vaccinations, which may vary along with clinical presentation, depending on the person affected.

## Clinical presentation

Babesiosis presents itself in different ways according to the clinical background of the patient (whether human or animal) and may be fatal in different populations, such as those that are splenectomised. Some studies suggest that *Babesia* infections are associated with a 40% mortality risk in humans; however, other research suggests that up to 20% is a more accurate mortality rate (in relation to *B. divergens*) in endemic areas for susceptible groups; this may be due to differences in the species of *Babesia* involved (Refs. 47, 31).

In humans, babesiosis is characterised by an incubation period of 1–6 weeks (following a tick bite) or 1–9 weeks (after transfusion), however, there have been cases where the incubation period exceeded the stated figures, for example, 175 days following a transfusion (Ref. 48). It is believed that the varied incubation period between individuals can be attributed to the immune status of the individual (Refs. 6, 49). Healthy individuals are commonly asymptomatic during infection; however, mild clinical signs will present in some patients, including flu-like symptoms such as fever, chills and headache. *B. microti* infections, for example, display a range of clinical presentations; some patients may be asymptomatic or have non-specific symptoms similar to the flu or in extreme cases can experience more serious presentations such as severe organ failure and death (Ref. 50). While healthy individuals usually recover, in patients categorised as ‘at-risk’ groups (i.e., the elderly, splenectomised or immunocompromised people), more severe symptoms such as haemolytic anaemia and splenomegaly may present, with infections eventually leading to fatality (Ref. 17).

TTB is a significant issue, with a mortality rate observed at ~19% (Ref. 51). *B. microti* has been observed increasingly in cases of blood transfusions (~70 to 100 cases of TTB over 30 years) and is now seen as the most common cause of TTB in the United States, highlighting the need for screening of blood samples before transfusions to avoid infection and potential serious complications, although the spread of other *Babesia* spp. via TTB varies (Refs. 36, 51). Previous research by Fang and McCullough covered 256 TTB cases, where 165 were positive for *Babesia* following a blood transfusion (Ref. 52). The number of TTB cases contributes to the recent global emergence of babesiosis and must be considered a much more serious issue (specifically in the United States), likely related to a wider distribution of vectors and reservoirs described previously. As individuals infected with *Babesia* may present asymptomatically and so may be unaware of infection, to continue donating blood for transfusions, further spread is likely. For babesiosis transmitted by other methods (i.e., direct contact with ticks and tick bites), the mortality rate is high at ~42% (for Europe), especially in splenectomised people (Ref. 53).

In tick-transmitted cases, severe illness such as organ failure or haemolytic anaemia is observed in ~58.5% of individuals; however, there is an overall low mortality rate of 1.6% (Ref. 54). Globally, a recent study reported a 2.23% mortality among the cases of human babesiosis included in the study; however, this meta-analysis was based on only 22 countries and 69 studies, so it is reasonable to suggest that this is a conservative estimate of the severity of infection (Ref. 16). Overall, despite the increased awareness of *Babesia* found in also human hosts, there are still limitations of its diagnosis due to a lack of *Babesia*-specific diagnostics for human use, and as such, global figures on mortality and reported symptoms are not readily available.

Interestingly, it is possible for humans to be infected with *Babesia* and other tick-borne diseases simultaneously. *B. microti, Borrelia burgdorferi* and *Anaplasma phagocytophilum* are all transmitted by black-legged ticks (*Ixodes scapularis)* and therefore co-infection is likely and has been reported. Co-infection is most notable in the United States, where babesiosis is more prevalent; however, it is also seen in Europe, for example, with co-infections resulting in Lyme disease and babesiosis present in Sweden (16.3% positive), Germany (11.5% positive) and the United States (14.1% for three human studies) (Ref. 55). When co-infection occurs, the severity and duration of clinical symptoms may be affected, where individuals may present with aggravated symptoms in comparison to infection with only one parasite type. This may make diagnosis and treatment more difficult, as issues may arise in terms of parasite identification. Splenectomised and immunocompromised patients experiencing fever should be considered for a confirmatory diagnosis of babesiosis, and patients should be tested at least using microscopy (e.g., blood films) as a minimum, but PCR and serology are also recommended for the diagnosis of babesiosis (Refs. 56, 57, 58). Rapid diagnosis of babesiosis, particularly in immunocompromised patients, is essential to ensure swift treatment courses where possible.

## Transmission and life cycle

**Figure 3.**
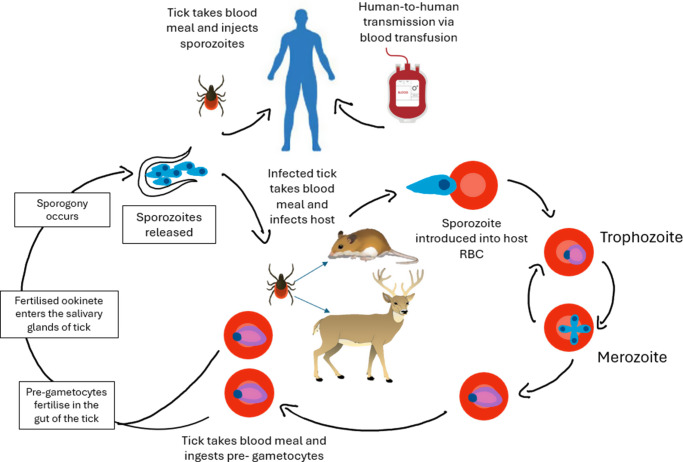
A simplified diagram of the life cycle of the *Babesia* parasite. A tick takes a blood meal from an infected host (such as the white-tailed deer or white-footed mouse) and ingests the sexual form of the parasite, pre-gametocytes. In the tick, these develop into gametes, which eventually fuse to form motile ookinetes and then penetrate the tick gut wall; here, a meiotic division takes place, which gives rise to kinetes that eventually travel to the hemolymph. Invasion of tick tissues, including the ovaries in the female tick or tick embryos follows with some kinetes also travelling to the salivary glands, where they develop into sporoblasts and eventually give rise to 5,000–10,000 infective sporozoites. The infected tick then takes a blood meal from a reservoir host or a dead-end host (humans), injecting sporozoites into the host, which invade RBCs; within the RBCs, the parasites develop into trophozoites (feeding stage) and eventually, merozoites (merogony). Merozoites are released from the RBC, and the cycle is repeated until the sexually distinct forms are also generated, the pre-gametocytes. These are ingested by feeding ticks and the cycle of infection continues in the arthropod host. Infection of humans may be through direct tick transmission or by blood transfusions with contaminated blood.

*Babesia* (both larval and nymph stages) require a mammalian reservoir host (from small mammals or birds to larger mammals such as deer) and a tick host (e.g., *I. scapularis*) to complete their life cycle, which typically lasts around 2 years (Ref. 17). Tick larvae feed on a vertebrate host after hatching (end of summer), ingesting parasites from an infected host. The larvae moult into nymph stages across winter, when the parasite gametocytes will move into the tick gut epithelium (after sexual development and replication takes place, followed by sporogony), with sporozoites travelling to their salivary glands. The tick nymph stages will once again feed, mature, and they can transmit *Babesia* to the next host (typically, human infection will take place at this stage as a zoonotic event). Adult stages of the tick will commonly feed on a mammalian host, such as white-tailed deer (Ref. 58). Within a mammalian host, including humans, *Babesia* parasites then infiltrate the RBCs, divide asexually via merogony and then exit to invade further RBCs. Sporozoites (released from the tick via its blood meal into the mammalian host) will begin the erythrocytic cycle, where trophozoites are formed intracellularly, which divide asexually via budding. Trophozoites develop into merozoites, continuing the erythrocytic cycle. Eventually, sexually distinct forms of the parasites are also formed, known as pre-gametocytes, which are ingested by ticks after a further blood meal, where the sexual cycle of the parasite is initiated (Ref. 34). The parasite can spread to thousands of offspring from only one maternal tick through a process known as transovarial transmission, whereby one female tick gives rise to thousands of eggs, disseminating *Babesia* parasites to said offspring (Refs. 15, 47, 59). This means that the parasite can spread rapidly to large numbers of ticks without the need to undertake a blood meal from an infected host, thus increasing the likelihood of a continuation of the infective cycle. A simplified schematic of this complex life cycle is shown in Figure 3.

**Figure 4.**
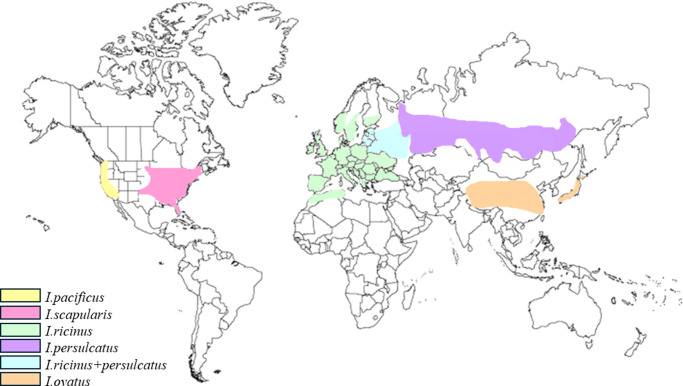
Geographical global distribution of different tick species. The presence of various tick species governs both the distribution of *Babesia* spp. and cases of human babesiosis across the globe (Refs. 8, 60).

Although the main vector for *Babesia* transmission is the *Ixodes* tick, other genera may be involved, such as *Dermacentor* ticks, as the species of tick may differ between *Babesia* spp. *B. microti* is transmitted by *I. scapularis*, observed to have a lifespan of 2 years. On the other hand, *B. divergens*, *B. venatorum* and *B. microti* are transmitted by ticks known as *I. ricinus* (Ref. 60). The ticks reported to transmit *B. duncani* are *Dermacentor albipictus* (*D. albipictus)*, and *B. crassa*-like variants are usually transmitted via *I. persulcatus* (Ref. 8). The global distribution of various tick species is shown in Figure 4; the distribution of ticks is related to the different species of *Babesia* found in similar geographical areas. The area of habitation and therefore, the species of tick may be relevant when diagnosing patients, as some diagnostic methods are effective for some *Babesia* spp. but not all.

## Diagnosis

Research suggests that *Babesia* infections are not well reported in Europe (or globally) and symptoms are poorly recognised, in addition, the actual number of cases may be significantly higher than indicated in the literature. This lack of information is of particular relevance for a swift diagnosis and treatment of human patients to avoid poor outcomes in particularly vulnerable patient groups (Ref. 61).

### Current diagnostic practice

In patients with high parasitaemia and suspected *Babesia* infections, microscopic examination of blood films to identify *Babesia* spp. is recommended. The microscopic examination of blood films is also historically the first diagnostic tool to be developed; blood films are stained with Giemsa or Wright stains and analysed by microscopy to detect the presence of *Babesia* spp. within the RBCs (Ref. 62). This requires a moderate level of training and can be done quickly; that is, blood smears and staining are complete within 30 minutes, meaning it is an effective diagnostic technique in medical emergencies, especially in cases where laboratories lack facilities to perform PCR. Identifying *Babesia* infections with associated low parasitaemia, however, poses a significant challenge, as the majority of babesiosis cases are asymptomatic or mild, and thus blood films may not be useful in these circumstances (Ref. 53). In addition, during the early stages of infection or in patients who have competent immune system, parasitaemia is low, and *Babesia* spp. are unlikely to be detected. In these cases, PCR is the preferred way to diagnose current infections, although its limitations due to as-yet-unknown or newly discovered species are highly relevant, particularly in recent years with new *Babesia* spp. periodically emerging in humans. Typically, diagnostic PCR uses *Babesia* spp.-specific primers and sequencing; however, issues arise when unexpected or rare species are present, so universal primers for all *Babesia* spp. should be used, followed by specific primers to allow species identification (Ref. 27). The most common primers for PCR have been described as covering a broad range of *Babesia* spp. using parts of the 18S rRNA gene, showing success in detecting a variety of species of *Babesia*, including those that are rare, such as the EU1 *Babesia* genotype (Ref. 63). Universal primers are also nowadays designed to target a specific consensus sequence of the internal transcribed spacer one gene of the 18S rRNA of the *Babesia* spp., as previously described (Ref. 64). As such, these universal primers have shown effectiveness where routine PCR provides negative results (Ref. 27).

Microscopic examination of blood smears and PCR are the most common methods of direct diagnosis of human babesiosis. There has been a distinct lack of advancement around diagnosis, such as the development of specific rapid diagnostic dipsticks or similar technologies developed for other parasitic infections, including malaria, and the initial recommendations for diagnosis of human babesiosis remain the same since their first mentioning/introduction in the literature in the 1980s (Ref. 65).

### Serological methods

In addition to PCR and blood smears, specific antibody-based assays may become useful, which rely on detecting parasite-derived proteins, such as parasite surface antigens, for example, by indirect fluorescent antibody testing, but issues arise when specific parasite surface antigens are not well characterised or documented, or there is an overall lack of antibodies to such antigens. Studying these antigens may provide the basis for vaccination development and/or biomarker identification for monitoring progress during treatment, for example (Ref. 66).

Immunofluorescence assays (IFAs) and enzyme-linked immunosorbent assays (ELISA) have some use in detecting and diagnosing babesiosis, but they lack sensitivity against a range of *Babesia* spp. In addition, IFA can also detect antibodies in serum samples from potentially infected individuals, indicating a past infection. *Babesia* antigens are incubated with patient serum samples containing antibodies and then reacted with immunofluorescent tags. Fluorescently tagged antigens (via binding by the antibodies in the sample) are detected using fluorescence microscopy, with fluorescent signals indicating the presence of antibodies to the parasites in the serum. These IFA tests IFAT are useful when parasites cannot be detected in blood smears; however, serological responses to infection may take up to 3 weeks, and therefore, they are only applicable to chronic infections or confirmation of past infections or exposure to parasites (e.g., caused by *B. microti*) (Ref. 67). As such, serology is more useful for retrospective analysis at the species level. Overall, however, microscopic blood film analysis is still considered the gold standard for serological testing of babesiosis (Ref. 68).

Where IFA testing has shortcomings, ELISA-based testing may be more useful, using antigens isolated from *Babesia* spp. For example, the recombinant merozoite surface antigen 1 for *B. bovis*, which has been used successfully to detect infections in cattle, yielded a sensitivity of 87% and a specificity of 80% by indirect ELISA (Ref. 68). An ELISA-based method for testing human blood for the presence of anti-*B. divergens* immunoglobulin G (IgG) antibodies has also recently been developed (Ref. 69). This test used merozoites as antigens from the parasite to detect antibodies and showed success in humans, with high sensitivity and specificity (86% and 100%, respectively) in comparison to other studies using different antigens from *B. divergens* or *B. microti.* Antigen capture assays (ELISA-based) specifically for *B. duncani* have been recently established, with reported high sensitivity (e.g., can detect 115 infected RBCs per μl) and specificity (no cross-reactivity from other species of *Babesia*) (Ref. 70). These assays have huge potential in the diagnosis of human babesiosis, including for use in point-of-care (PoC) testing, specifically if further testing can be achieved to include a wide range of *Babesia* spp. Limitations to the use of ELISA include the number of time-consuming steps required to provide a diagnosis. In addition, these tests seem to be less sensitive and specific than other methods, such as IFA. Also, the need for sophisticated equipment for data readout and interpretation highlights the demand for novel and easily accessible rapid diagnostic techniques (Ref. 71).

### Emerging diagnostic techniques

Immunochromatography-based lateral flow tests are one example of novel diagnostic tools for the improved diagnosis of *Babesia* infections. A rapid, bovine-specific immunochromatographic test (ICT) was developed, using the recombinant C-terminal portions of the rhoptry-associated protein-1 of *B. bigemina*, and the recombinant merozoite surface antigen-2c for *B. bovis* as antigens on test strips for simultaneous diagnosis of the two species. This test showed promising results, establishing a diagnosis in under 15 minutes, without the need for trained personnel to perform the test (reducing time, cost etc.) and demonstrated high sensitivity and specificity (96.7% and 91–93%, respectively) in diagnosing both *B. bigemina* and *B. bovis;* however, human species were not tested, so further exploration is necessary (Ref. 72). More recently, ICT tests were trialled by other researchers based on the previous study. These tests can also be used on-site and only require 5–30 minutes for a result. ICT strips used *B. bigemina* RAP-1/CT17 and *B. bovis* SBP-4 as detection antigens on a nitrocellulose strip and were tested with bovine serum samples and displayed success, for example, in detecting antibodies for *B. bovis*, where even PCR was not successful. These tests are seen to be more useful than ELISA and IFAT, due to their ease of use, and they show promising results for PoC testing (POCT) for bovine babesiosis. However, so far, they cannot be used in diagnosing humans (Ref. 73). Furthermore, a lateral flow test with recombinase polymerase amplification (RPA) was developed, using the mitochondrial cytochrome oxidase subunit I gene of *B. microti* (Ref. 74). This test had promising results, allowing for the detection of as few as 0.25 parasites/μl blood (40× more sensitive than standard PCR), with no cross-reactivity with other *Babesia* spp. This test was quicker (10–30 minutes) and easier to perform than standard PCR, as it is more stable and can be performed at a range of temperatures between 25 and 45°C; however, the lateral flow test cannot be performed without first completing RPA, which requires an extra 10–30 minutes, and this method was only trialled on *B. microti* infections. Development of a lateral flow device for use in humans without the need for a laboratory, based on high sensitivity and specificity for a range of different *Babesia* spp. would aid in the rapid diagnosis of infected individuals, allowing for swift treatment, the implementation of prevention measures and the avoidance of areas where reservoirs show high infection rates. Thus, by continuing research into *Babesia* species in terms of antigen identification for inclusion in such diagnostic setups, the production of similar capture assays and rapid detection should be achievable in the near future.

One other study details the development of an antibody capture assay using novel monoclonal antibodies and the detection of BmGPI12 from the plasma of infected humans (this is an important antigen secreted by *B. microti* into the human body amidst infection). This assay (mGPAC) proved successful, and sensitive in comparison to other methods of diagnosis, which may not be reliable when parasitaemia is low, it requires only 1 μl of plasma, so it has the potential to be useful in cases where high-throughput screening, such as in screening blood samples for blood transfusions, is required. However, the test specificity or range of species detected has so far not been established; therefore, further testing on infections with different *Babesia* spp. should be considered (Ref. 75). Other studies have been completed using this same antigen for additional development of ELISA-based antigen capture assays (e.g., BmGPAC, with high sensitivity (20 pg of BmGI12 per μl blood)), along with rapid detection, proving that this antigen is extremely useful in detecting *B. microti* infection in human sera samples; therefore, similar testing and methods should be completed on antigens from other *Babesia* spp. that infect humans (Refs. 76, 77).

**Figure 5.**
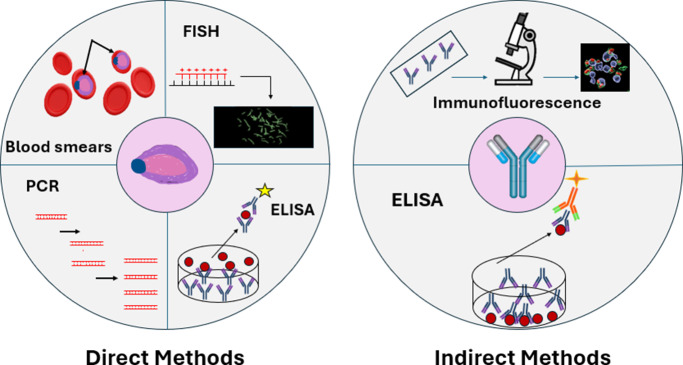
Overview of commonly used and recently developed techniques to diagnose recent and past *Babesia* infections by direct and indirect methods. Direct detection is achieved by various methods, including in blood smears, by FISH, PCR and ELISA whereas indirect detection relies on the detection of antibodies to the parasites using IFA and ELISA (Table 1).

Finally, research carried out within the last 5 years has seen the development of a *Babesia*-genus-specific fluorescent in situ hybridisation (FISH) technique (Ref. 78). This test has been demonstrated to be useful in diagnosing *Babesia* infections in humans, with relevance for a variety of human-important *Babesia* spp., and is also able to distinguish *Babesia* from similar tick-transmitted parasites such as *Borrelia* spp*.*, making it especially useful for individuals experiencing co-infection. In addition, it can detect the parasite at low parasitaemias (~0.001% for *B. microti* and *B. duncani*) and can be conducted in the laboratory, without the need for expensive equipment, providing a result within 2 hours. The test targets *Babesia* 18S rRNA, and the parasites, if present, fluoresce green and are therefore easy to detect visually. FISH appears to be the most useful diagnostic technique in terms of detecting *Babesia* spp. this far but still has drawbacks in terms of time to perform the assay and the need for a laboratory setup. Further developments are required to enable on-site diagnosis of human babesiosis, for example, through the use of a lateral flow test suitable for humans, capable of detecting all species of *Babesia*, to improve treatment responses and improve patient outcomes. A graphical summary of the most commonly used techniques is given in Figure 5.

**Table 1.** Summary of direct and indirect detection of human babesiosis

Test	Used in humans	Detection (direct or indirect)	Advantages	Disadvantages
Peripheral blood smear (Giemsa or Wright stain)	Yes	Direct Microscopic examination for presence of parasites in RBCs; (checked for distinct features such as “Maltese cross” formations, for example, in *B. microti* infections)	*Sensitivity:* 50–70% (at high parasitaemia) Rapid (30 min including smear and microscopic examination) Low cost Useful at height of infection (i.e., high parasitaemia) Distinct appearance of parasites in their blood stage	Low sensitivity (especially at low parasitaemia) Requires skilled technician May miss co-infections
PCR (polymerase chain reaction)	Yes	Direct Direct detection of *Babesia* DNA in blood sample	*Sensitivity:* high (>90%) and specific Can detect low parasitaemia Useful for species identification	Expensive Not widely available in all settings Requires specialized equipment and laboratory setting
Serology (IFAT – Indirect fluorescent antibody test)	Yes	Indirect Detects antibodies (IgG, IgM) against *Babesia*	*Sensitivity:* Varied (88–96%) depending on stage of infection when sampled *Specificity:* High (up to 100% in patients with other tick-borne diseases) Useful for retrospective or past infection Can help confirm diagnosis when microscopy is inconclusive	Cannot distinguish current vs. past infection Antibodies may take 1–2 weeks to develop Some cross-reactivity with other parasites (e.g., malarial parasite)
Real-time PCR (qPCR)	No	Direct Quantitative detection of *Babesia* DNA	*Sensitivity:* High Can quantify parasite load Useful in treatment monitoring	High cost Not routine in all labs Requires specialised equipment
FISH	No	Direct Detects parasites in blood smear by hybridization of fluorescent probe specific for parasite spp.	*Sensitivity:* High (at height of parasitaemia) Rapid: Less than 2 hours *Specificity:* Can differentiate between species, for example, *B. microti* and *B. duncani* Complementary to other diagnostic methods	Requires trained personnel and specialised equipment Not as sensitive as PCR May give false negative result at low parasitaemia
Nucleic acid amplification tests (NAATs)	No	Direct Molecular assays that amplify *Babesia* DNA	*Sensitivity:* High May be used in blood donor screening	Limited availability High cost Need for specialised equipment

## Treatment and prevention

Timely treatment of babesiosis is essential for those who are immunocompromised; otherwise, the infection is likely to be fatal. There are several different methods of treatment that are authorised for use in cases of human babesiosis, but they all have their respective drawbacks. Treatments for humans, in general, are based on experiences from treatments of animals.

For animals, such as dogs, the recommended treatment for babesiosis infection is diminazene aceturate or imidocarb dipropionate, but these are not authorised for use in humans due to the emergence of resistance, toxicity and negative side effects upon discontinuation of courses (Refs. 79–80). However, two cases of human babesiosis in Ireland have been successfully treated for *B. divergens* infections with imidocarb, recommended for use in animals, suggesting that this particular compound requires further experimentation and consideration for use in humans (Ref. 81). For humans presenting with mild to moderate clinical symptoms (e.g., flu-like symptoms), babesiosis, mainly through infection of *B. microti*, is treated with a combination therapy of atovaquone and azithromycin (Ref. 82). Atovaquone and azithromycin are both antibiotics that have broad-spectrum activity together, making them effective against several infectious agents, including *Babesia* (Refs. 83–84). There is cumulative evidence that Atovaquone, in particular, efficiently clears *B.* *divergens* infections (Ref. 64). For more severe cases, the recommendation is treatment with clindamycin and quinine (Ref. 17). Clindamycin is a lincosamide antibiotic that works via inhibition of bacterial protein synthesis at the 50S ribosomal level, thereby decreasing the production of toxins and increasing microbial opsonisation and phagocytosis (Ref. 85). There are opposing studies determining the effectiveness of Clindamycin for severe babesiosis, with a combination of azithromycin and atovaquone being suggested instead of current recommendations, as they may be unsuccessful (Refs. 86–87). Quinine is an antimalarial compound of which the mode of action against *Babesia* is not entirely known, but research suggests it may inactivate biological functions in specific organelles in *Babesia* spp., such as the mitochondrion (Ref. 50). Quinine is believed to have limited efficacy against babesiosis as a monotherapeutic and does not facilitate the clearance of the parasite. Combining clindamycin with quinine shows better reduction of parasitaemia levels than clindamycin alone. However, the negative side effects associated with this combination (quinine is especially not well tolerated), such as hearing loss, vertigo and tinnitus, may outweigh the response for the patient; side effects may also affect adherence to the course of drugs given (Table 2). Therefore, a combination of these therapies, without the addition of quinine, is suggested to be more effective in treating babesiosis, while avoiding the most severe side effects reported following quinine administration (Ref. 88). It is worth noting that each of these described drugs were not specifically formulated for babesiosis treatment; instead, they are used due to previous successes in treating other conditions such as toxoplasmosis (atovaquone) and bacterial infections (azithromycin) (Ref. 50).

**Table 2.** Summary of most common treatments for human babesiosis

Treatment	Mechanism	Advantages	Disadvantages
*Atovaquone* (750 mg 2× a day (adults), 20 mg/kg 2× a day (children))	Antimicrobial compound; inhibits mitochondrial transport via the cytochrome bc_1_ complex, and ATP synthesis.	Broad-spectrum activity. Works well in combination with azithromycin.	Not specific for babesiosis. Results in severe side effects. Antibiotic resistance.
*Azithromycin* (500–1000 mg first, then 250–1000 mg daily (adults), 10 mg/kg then 5 mg/kg daily (children))	Antibacterial; inhibits bacterial protein synthesis and pro-inflammatory cytokine production etc.	Broad-spectrum activity in combination with atovaquone.	Not specific to babesiosis. Severe side effects. Antibiotic resistance.
*Clindamycin* (600 mg 3× daily (adults) 7–14 mg/kg three times daily (children))	Lincosamide antibiotic; inhibits protein synthesis at 50S ribosome level.	Works in combination with quinine to reduce parasitaemia.	Ineffective in severe cases of babesiosis. Not effective when used alone. Severe side effects possible.
*Quinine* (650 mg 3× daily (adults) 10 mg/kg (children))	Anti-malarial compound, mechanism in babesiosis unknown.	Useful in reducing parasitaemia when combined with clindamycin.	Severe side effects possible. Shown to be highly toxic. Limited when used alone.

In individuals suffering from severe symptoms, a high parasitaemia (above 10%) and poor prognosis, exchange transfusion may be used instead of traditional treatment courses to replace the infected blood cells with new ones and remove toxic metabolites, allowing for a rapid reduction of parasitaemia (Ref. 82). Antimicrobial interventions are then administered as they display increased efficacy following exchange transfusion (Refs. 88–89). Typical treatment spans 7–10 days, with high doses combining the drugs mentioned previously; however, side effects are frequently reported, along with some clinical cases of recurrence; these are seen to depend on the dose of drugs given and the treatment duration. With prolonged treatment and higher doses, drug resistance becomes more likely and has recently been reported in cases of *B. microti.* Occasionally, discontinuation of the course is recommended in the presence of severe side effects (Refs. 50, 90). Current treatment options are lacking in areas that have been described above, highlighting the need for continued research in this area.

### Novel treatment developments

New drug candidates for the treatment of human babesiosis include clofazimine, trans-chalcone, nitidine chloride (NC) and 17-dimethylaminoethylamino-17-demethoxygeldanamyci (17-DMAG). However, these drugs have only been tested for the preclinical phase of babesiosis infection, meaning their efficacy in the clinical phase is unreported. Clofazimine is a compound with antimycobacterial properties, used for several conditions such as tuberculosis. A combination of atovaquone with clofazimine is seen to be effective not only in preventing parasitaemic rise but also in achieving a radical cure of babesiosis, something that is impossible with the combination of atovaquone and azithromycin. However, testing remains confined to animal models, so further research is required to determine potential efficacy in humans (Ref. 91).

Chalcones are natural products of the flavonoid family, used for their broad-spectrum biological activities, for example, as anti-malarials. These inhibit the bc_1_ complex similarly to atovaquone, potentially explaining their efficacy in treating babesiosis. Trans-chalcone (TC) is seen to inhibit multiplication and growth of *Babesia* spp. *in vitro*; however, the exact mechanism is unknown. *In vivo* studies in mice showed positive results for the use of TC, especially when combined with clofazimine or atovaquone. Further research is necessary to determine how these compounds react to human babesiosis (Ref. 92). NC is a topoisomerase inhibitor, used to disrupt the process of DNA transcription. NC is known to possess anti-inflammatory properties, among other effects and was shown to be effective against *B. caballi* and other *Babesia* spp. seen in animals, but lacks reporting in human-infecting *Babesia* spp. Thus, further exploration of NC is necessary to determine if it is effective against other species (Ref. 93). 17-DMAG is an analogue of geldanamycin (an inhibitor of a drug target known as Hsp90). *In vitro* culture and mouse models demonstrate that this compound can inhibit the multiplication of *Babesia*; combining 17-DMAG and atovaquone showed effectiveness in treating babesiosis in mouse models (Ref. 80). As a result, 17-DMAG provides a potential option for treatment in animals and humans, but its efficacy against other *Babesia* spp. and safety in humans must be determined first.

As reported, drug-resistance is still an issue for these new candidates as high doses are necessary for parasite clearance, meaning they may not be useful for a prolonged amount of time, and more compounds should be explored (Ref. 90). Other new drug candidates include a family of drugs known as 6,7-dimethoxyquinazoline-2,4-diamines (DMQDAs), following a study on 20 compounds that revealed 14 exerted anti-babesial activity. One such compound is SHG02, which showed high levels of anti-babesial activity when compared to a control group (working on *B. rodhaini*). The direct mechanism of this compound in relation to *Babesia* clearance is not well known, and these studies were completed *in vitro* using mouse models, so they may not accurately represent human interactions. Follow-on studies should be completed on these compounds, particularly SHG02, to determine their mode of action and efficacy against a range of *Babesia* spp. in humans (Ref. 94).

Tafenoquine (TAF) has been discussed as a potential treatment option for babesiosis, as it has previously been utilised for the treatment of other blood-related diseases such as malaria, with proven broad-spectrum activity against protozoan parasites (e.g., *Toxoplasma gondii*) (Ref. 92). One thing to note about using TAF is the negative side effects experienced by those who present with a deficiency of glucose-6-phosphate dehydrogenase (G6PD), namely causing severe haemolytic anaemia. Recent research highlighted that it is possible to use TAF to control canine and feline babesiosis with a single dose (10 mg/kg), along with being used to prevent bovine, ovine and equine babesiosis, suggesting that it could be useful in cases of human babesiosis. A combination of TAF and atovaquone is seen to be useful in curing models of human babesiosis in animals, with no disease relapse. While effective, the direct mechanism by which TAF works is still unclear (Refs. 50, 95). To corroborate these findings, further research has stated that TAF is effective in clearing *B. microti* infections in hamsters and mice (including immunocompromised mice), demonstrating a clear potential use of TAF in clearing *Babesia* from infected humans, which is clinically relevant for immunodeficient patients, as many who experience severe symptoms from a babesiosis infection are (Ref. 96). Despite these positive responses to TAF, it is still not approved by the FDA for use in the treatment or prevention of human babesiosis (Ref. 97).

While several new targets are discussed here, it seems that (at least in European countries), the combination of azithromycin and atovaquone or clindamycin and quinine is still the most widely recommended treatment for babesiosis in humans (Table 3). Companies such as 60 Degrees Pharmaceuticals are currently undergoing selection of candidates for a Phase II clinical trial of TAF in babesiosis patients (double-blind, placebo-controlled; NCT06207370) (Ref. 98). TAF and SHG02 are two drug candidates that have shown promise and should therefore be considered for use in humans.

**Table 3.** Summary of new developments in babesiosis treatment

Treatment	Mechanism	Advantages	Disadvantages
Clofazimine	Antimycobacterial compound. Mechanism not well reported.	Useful in combination with atovaquone (potential radical cure).	Human use not reported, efficacy in humans is unknown. Only preclinical evidence for potential efficacy.
Trans-chalcone	Similar action to atovaquone. Broad-spectrum biological activities.	Inhibit multiplication and growth of *Babesia* in vitro.	In vivo research only on mice, human interactions unknown, only preclinical studies.
Nitidine chloride	Topoisomerase inhibitor disrupts DNA transcription.	Effective against several *Babesia spp.* in animal models.	Lack of studies in humans, so unknown interactions, no clinical trials as yet.
17-DMAG	Inhibitor of Hsp90 (analogue of geldanamycin).	Inhibition of *Babesia* multiplication. Effective in treating babesiosis in mice models, in combination with other drugs.	Lack of studies in humans, unknown interactions, promise shown in preclinical studies.
DMQDA’s	Direct mechanism unknown.	High levels of anti-babesial activity.	Only animal models, unknown responses in humans.
TAF	Broad-spectrum activity against protozoans.	Single dose to treat canine and feline babesiosis. Combining TAF and atovaquone shows cure with no relapse in animal models.	G6PD deficiencies result in haemolytic anaemia. Not approved by the FDA for human use. Only models of human babesiosis in animals – may not be representative. Clinical trials beginning soon.

Further exploration of potential treatments for babesiosis is essential, as the recommended treatments produce many adverse effects and their use contributes to antibiotic resistance. Narrow-spectrum antibiotics made specifically for the treatment of babesiosis would be more useful to avoid this potential resistance emerging, as there is currently no babesiosis-specific treatment available.

Alongside treatment advances, vaccinations are also in development to help protect against babesiosis when living in or travelling to endemic areas. Vaccination becomes complex due to the need for protection against the different developmental stages of *Babesia* to ensure immunity (e.g., sporozoites and blood-stage parasites). Research on vaccines is extensive, covering a variety of different types. Non-living vaccines (killed parasites or exoantigens) have previously demonstrated effectiveness against *B. bovis*, for example. Soluble parasite antigens have shown use in experimental vaccinations, protecting cattle against tick infection (with promising results against *B. bigemina* and *B. canis*). Recombinant subunit vaccine development exhibited some success (against *B. canis*) but also varies in protection. However, only a small number of antigens have been tested for subunit vaccines, and these were tested in mouse models, which need further validation in clinical trials (Ref. 99). A promising avenue for vaccination is through transmission-blocking vaccines, which may be feasible, as evidenced by previous successful developments against *Plasmodium.* Currently, a successful vaccine for human use is not available, but there are many potential areas to explore that can provide useful results for a vaccine that could protect against multiple stages of both *Babesia* development and *Babesia* spp. (Refs. 100–101). A whole-parasite vaccination has previously shown promise in splenectomised animals and could lead the way for a whole-parasite vaccine useful against human babesiosis (Refs. 98, 102).

In addition, tick antigens have been explored for the development of vaccines, which may prove more useful than acaricides for controlling tick populations and therefore may be effective in reducing *Babesia* in humans and animals alike. While promising, some concerning issues remain, including polymorphisms and cost (Refs. 103–105).

### Future direction

There has been an increase in research on human babesiosis in recent years, and it can be anticipated that interest in this disease will increase with increasing numbers observed globally. Advancements have been made in identifying, diagnosing and treating human infections; however, these areas are still underdeveloped in many ways, with a demand/need for species-specific and sensitive rapid diagnostic tests, vaccines and drugs designed especially for human infections. The global economic impact of human babesiosis is difficult to ascertain, and there remains a serious lack of information regarding surveillance of tick vector distribution, along with the impact of climate change on babesiosis, as current research suggests that this affects tick vector distribution and therefore influences the incidence of babesiosis in humans (Ref. 17). Increased global awareness of human babesiosis is vital to facilitate effective disease management and prevention, as the disease may be fatal in certain patient groups. Continued epidemiological surveillance is necessary to provide updates on emerging species, as *Babesia* is evolving, indicated by the occurrence of human infections by animal species and increasing incidences of antibiotic resistance.

Diagnostics have improved over the years with especially the advent of PCR technology, but there is still a distinct lack of rapid diagnostic tests as POCT for application in humans. Recent diagnostic assays have shown improvement over traditional methods (such as blood smears) in terms of sensitivity but still have their own limitations. IFA, ELISA and FISH methodologies show promise in effectively diagnosing babesiosis, but each method requires specialist training, equipment and time. Ideally, diagnosis should be rapid and without the need for specialist personnel or equipment, promoting test efficiency at a low cost. A rapid ICT has shown promise in diagnosing bovine babesiosis, and fits the aforementioned requirements; as such, it follows to adapt this test format to detect human-infecting *Babesia* spp. Development of antigen capture assays and ELISA-based tests has proven that there is potential for sensitive and specific laboratory-based diagnostic tests, based on monoclonal antibodies to species-specific antigens, such as those developed for *B. microti.* Lateral flow devices, which are cheap to produce and give a result within minutes, have been described for use in animals, and research suggests that this is a potential avenue for exploration for human babesiosis. Development of a lateral flow device should include useful components from other methods (such as monoclonal antibodies) to establish an accurate, specific and rapid test covering a range of human-infecting *Babesia* spp.

Treatment of babesiosis has been widely explored, yet recommendations have not changed for many years, despite individuals experiencing severe adverse effects. Resistance to treatment is also on the rise, making these treatment options less effective in patients; therefore, research into new treatment options is vital to provide relief from symptoms and reduce babesiosis-associated mortality. The novel treatment options described in this review suggest promising options for treating human babesiosis more effectively than traditional methods; TAF and imidocarb, for example, show high levels of success in animal models but require additional experimentation via clinical trials to determine if the observed effects are mirrored in humans before they can be incorporated in the standard treatment regime for babesiosis.

As of now, there is no vaccine for the prevention of or protection from *Babesia* infections; many avenues are being explored, of which several have shown promise for development into human-safe vaccines. Antigen studies have identified potential antigens for a target in subunit vaccine development, and whole-parasite vaccinations may be possible, with success shown in splenectomised animal models. Vaccine development requires continuation to produce an effective vaccination capable of targeting multiple *Babesia* spp. and various stages of development, useful both before transmission and during blood-infection. Potential vaccines should be used in endemic areas, especially for high-risk individuals. While vaccine prevention is not yet possible, eliminating or reducing interactions between humans, ticks and/or the vectors of *Babesia* could reduce the number of babesiosis cases. Addressing small-scale issues, such as proximity to vectors, could help to prevent transmission or eliminating the vector tick through the use of acaricides to reduce infections. Finally, increased public awareness of tick-borne diseases, such as babesiosis, should be promoted by governmental initiatives, especially in endemic regions, to lower the overall risk of exposure to tick vectors and infection in humans.
